# Risk of acute coronary syndrome and relationship with the use of khat and tobacco products in the Jazan region, Saudi Arabia: A prospective case-control study

**DOI:** 10.18332/tid/189950

**Published:** 2024-07-08

**Authors:** Rashad Alsanosy, Mohamed Salih Mahfouz, Abdulelah Mohammed Arishi, Siddig Ibrahim Abdelwahab, Manal Taha, Osama Albasheer, Hasan Mohammed Daghriri, Khalid Ahmed Majrashi, Abdullah Farasani, Ahmed A. A. Altraifi, Isameldin E. Medani, Nasser Hakami

**Affiliations:** 1 Department of Family and Community Medicine, Faculty of Medicine, Jazan University, Jazan, Saudi Arabia; 2 King Faisal Specialist Hospital and Research Centre, Riyadh, Saudi Arabia; 3 Health Research Centre, Jazan University, Jazan, Saudi Arabia; 4 King Fahad Medical City, Riyadh, Saudi Arabia; 5 Department of Medical Laboratory Technology, College of Applied Medical Science, Jazan University, Jazan, Saudi Arabia; 6 Obstetrics and Gynecology Department, College of Medicine, Jazan University, Jazan, Saudi Arabia; 7 Surgical Department, College of Medicine, Jazan University, Jazan, Saudi Arabia

**Keywords:** acute coronary syndrome, khat chewing, tobacco, risk factors, Saudi Arabia

## Abstract

**INTRODUCTION:**

Previous studies have identified several risk factors for acute coronary syndrome (ACS). This study was intended to examine the potential risk of ACS associated with khat and tobacco use.

**METHODS:**

A case-control study of 344 people (172 cases and 172 controls) was conducted at Prince Mohammed Bin Nasser Hospital in Jazan, Saudi Arabia, from April to September 2019. The cases and controls were matched for age (±5 years) and gender. Data were analyzed using descriptive, inferential, and modeling analyses. We utilized the adjusted odds ratio (AOR) to express the results.

**RESULTS:**

The prevalence of ever khat chewing among all study participants was 29.1%, significantly higher for the cases with ACS than for the control group (43.6% vs 14.5%, p<0.001). Cigarette smokers accounted for 33.4% of the study participants, and 22.1% were ACS cases, which is a significantly higher percentage than the control group. The prevalence of smokeless tobacco was 20.3% among ACS cases and 14.5% among controls, with no statistically significant differences (p>0.05). In the final model, tobacco use was more likely to be reported among cases with myocardial infarction (MI) (AOR=4.58; 95% CI: 1.01–4.73, p<0.05) as was khat chewing (AOR=3.4; 95% CI: 1.55–7.46, p<0.05), after controlling for other traditional risk factors.

**CONCLUSIONS:**

Khat chewing was reported more by those who reported ACS. ACS cases were more likely to be frequent khat users with chewing sessions of five or more days per week. Regular tobacco use was also reported in those who reported ACS, and this increases with the amount of tobacco used. Implementing early intervention strategies can help mitigate the impact of khat chewing and smoking on the development of ACS.

## INTRODUCTION

Acute coronary syndrome (ACS) is a potentially life-threatening condition in which ruptured atherosclerotic plaque triggers platelet aggregation with activation of the coagulation cascade, resulting in either partial or complete blockage of coronary flow. As a result of this blockage, the patient may present with unstable angina or myocardial infarction^1^. In Saudi Arabia, non-communicable diseases accounted for more than 70% of all causes of death in 2018, according to the World Health Organization (WHO)^2^. In Saudi Arabia, diabetes and hypertension were the most prevalent risk factors in ACS patients, reported in approximately 50% of cases^3,4^. Additionally, new emerging risk factors were discovered recently in multiple studies, which may share their attribution in developing future coronary artery diseases^5,6^.

Khat is a chewable green leaf addictive stimulant similar to amphetamine. It is widely consumed in East Africa and the Southern Arabian Peninsula, with a prevalence of 33.2% in Saudi Arabia’s Jazan region^7^. Khat consumption has been linked to elevated heart rate and blood pressure, increased risk of cardiovascular and cerebrovascular disease, and higher mortality in ACS patients. Khat chewing is an independent risk factor for myocardial infarction, with a dose-response relationship^8,9^.

Waterpipe smoking (WP) is common in the Jazan region; the estimated prevalence of current waterpipe smokers among Jazan University students in 2018 was 34%^10^. A systematic review in 2016 found that WP smoking adversely affects the cardiovascular system by causing hypertension, tachycardia, and increasing blood vessel resistance^11^. Waterpipe smoking has also been linked to an increased risk of coronary artery disease, an increased incidence of ST-elevation myocardial infarction (STEMI) compared to non-ST-elevation myocardial infarction (NSTEMI), an increased risk of re-infarction, and mortality^12,13^.

Smokeless tobacco (ST) (shammah) is a product of tobacco powder and other ingredients prepared to be taken by mouth, under, or at the sides of the tongue, and it is common among the Saudi population, Yemeni, and Sudanese citizens^14^. Shammah has been shown to harm the body^15^. There is conflicting data on whether ST has an actual impact on causing cardiovascular disease. Previous studies revealed an association between smokeless tobacco products and developing a life-threatening acute coronary event, myocardial infarction, or stroke^16,17^. In contrast, other studies did not show a significant association between ST and cardiovascular disease^18^.

Khat chewing habits, waterpipe smoking, and smokeless tobacco use are popular among the Jazan population^7,14^. Still, there needs to be more data in the Jazan region of Saudi Arabia that has studied the effect of these behavioral factors on coronary artery diseases. Therefore, we aimed to identify the common risk in the Jazan region attributed to ACS development and analyze the association of khat chewing, waterpipe smoking, and smokeless tobacco with acute coronary syndrome through a hospital-based prospective case-control study.

## METHODS

### Study design and setting

A prospective case-control study was conducted at Prince Mohammed Bin Nasser Hospital, the only governmental hospital in Jazan, Saudi Arabia, with a cardiology ward, coronary care unit, and cardiac catheterization center. It is the primary referral center for cardiac cases from other hospitals and primary care units in the Jazan region. Data collection occurred over six months, starting on 25 April 2019. Jazan Province is the second-smallest administrative region in Saudi Arabia, located in the southwest along the southern Red Sea coast, bordered by the Aseer region to the north and Yemen to the south. The province has a population of approximately 1.6 million^19^. The study was conducted in accordance with the Declaration of Helsinki, and approved by the Institutional Review Board of Jazan General Hospital (H-10-Z-068), Ministry of Health, Saudi Arabia (protocol NO. 1913; 2019). Informed consent was obtained from all the subjects involved in the study before data collection.

### Participant’s selection, diagnostic criteria, and sampling procedure

Cases were patients newly diagnosed with acute coronary syndrome (ACS) who were admitted to the coronary care unit or cardiology ward at Prince Mohammed Bin Nasser Hospital in Jazan, Saudi Arabia, from April to September 2019. ACS is diagnosed based on American College of Cardiology and American Heart Association guidelines, which require at least two of the following features: typical electrocardiographic changes, compatible clinical symptoms, and elevated cardiac biomarkers (troponin T and I)^20^. Cases and controls were matched on age (±5 years) and gender. Controls were preferentially selected from patients admitted to the same hospital for unrelated conditions, especially from dermatology, surgery, and internal medicine outpatient clinics. Control patients with any previous history of cardiovascular or cerebrovascular diseases were excluded, resulting in a total sample of 344 participants (172 cases and 172 controls), with a power of 80% and a 95% confidence level. The sample size for the case-control study was determined using G*Power software^21^, considering the desired statistical power, significance level, and expected prevalence of the exposure and outcome of interest in the control group. The case-to-control ratio is 1:1, and the hypothetical proportion of control with exposure is 15, with the most minor extreme adjusted odds ratio to be detected being 2.2 (AOR values >2.2 indicating a significant risk factor).

### Exclusion criteria

We excluded patients with a history of acute coronary syndrome within the previous six months to minimize the risk of confounding by residual ischemia or cardiomyopathy. We also excluded patients with uncontrolled congestive heart failure, severe renal impairment, active infection, malignancy, or pregnancy to ensure that the study population was as homogeneous as possible and to reduce the risk of bias. The classification bias was avoided by using standardized criteria, objective tests, complete records, and eliminating financial incentives.

### Data collection

Data were collected through patient interviews using a structured questionnaire administered by a well-trained nurse. The questionnaire collected risk factors for cardiovascular disease, including demographic and background characteristics, medical history (including family history of coronary artery disease in first-degree relatives), behavioral risk factors (khat chewing, smokeless tobacco, waterpipe smoking, and cigarette smoking), and medical risk factors (lipid profile, hypertension, diabetes mellitus, fasting blood glucose, waist–hip ratio, and physical activity). Central obesity defined as a waist-to-hip circumference ratio of ≥0.8 for women and ≥1.0 for men. Total cholesterol of >200 mg/dL is considered a high level. Serum triglycerides level is considered high when >150 mg/dL. LDL >130 mg/dL is considered a high level. HDL levels >40 mg/dL in men and >50 mg/dL in women are considered high. Fasting blood sugar level ≥126 mg/dL indicative of diabetes. Physical activity was categorized as low activity levels, moderate activity levels, or high activity levels. A high level of physical activity is considered when physical activity levels correspond to approximately one hour of activity per day or more. A moderate activity level when doing some activity is more than likely equivalent to half an hour of at least moderate-intensity physical activity on most days. When physical activity does not meet any of the criteria for either moderate or high level of physical activity, then it is a low level of physical activity. Information on medical risk factors was obtained from patient medical files. Missing data was handled using frequency matching.

### Statistical analysis

We used the Statistical Package for Social Sciences (SPSS) software to analyze the data. We used descriptive statistics, such as simple tables, frequencies, and cross-tabulations, to summarize the data. We used the chi-squared test to test for differences in proportions between groups and logistic regression models to estimate the risk or protective effect of each variable on acute coronary syndrome. The strength of the association between acute coronary syndrome and the predictors was measured by the adjusted odds ratio (AOR) and 95% confidence interval (CI). A p<0.05 was considered statistically significant.

## RESULTS

**Figure 1:**
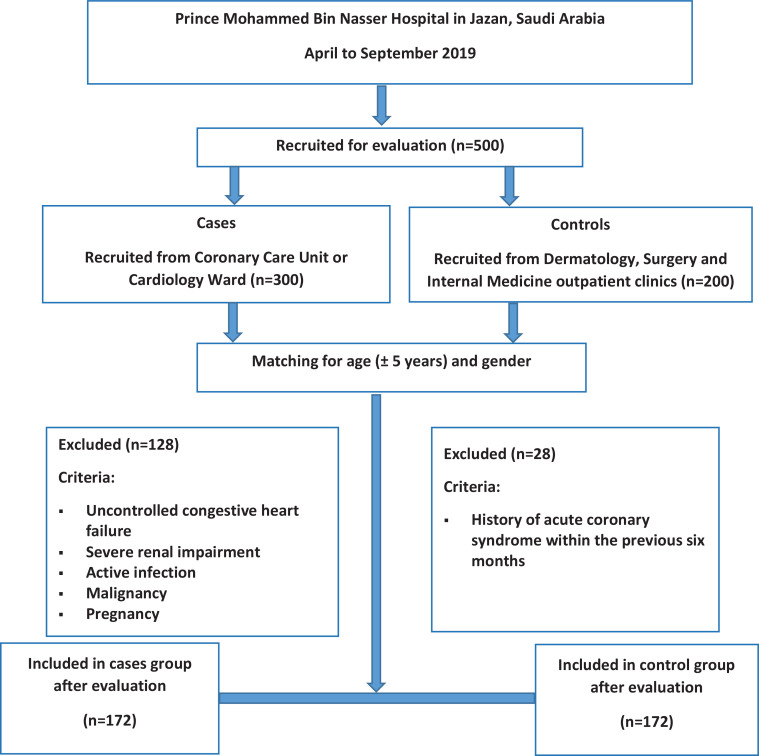
Flow diagram of participant selection for the cases and control groups, Jazan region, 2019

The flow diagram of this case-control study is presented in Figure 1. A total of 500 individuals were invited to participate in the study. Of these, 344 (68.8%) fulfilled the eligibility criteria, agreed to participate, and completed all of the study procedures. The remaining 156 individuals (31.2%) were excluded due to ineligibility criteria.

Table 1 compares cases and controls on demographic, socioeconomic, and selected clinical characteristics. Gender, age, urban residence, and income status were similar (p>0.05) between cases and controls. There were no differences between the case and control groups regarding mean age (p>0.05). In addition, there were no significant differences (p>0.05) between the duration and amount consumed of all substance use, except for the number of heads used for waterpipe. However, there were significant differences between the two groups in lipid profile (p<0.05 for all) and physical activity (p<0.001). Systolic and diastolic blood pressure and fasting plasma glucose were similar between the two groups.

**Table 1:** The basic sociodemographic and clinical characteristics of the case and control groups involved in the study of the relationship between the use of khat and tobacco products and the risk of acute coronary syndrome in the Jazan region of Saudi Arabia, in 2019 (N=344)^a^

*Characteristics*	*Cases n (%)*	*Controls n (%)*	*p*
**Gender**			
Male	124 (72.1)	124 (72.1)	0.999
Female	48 (27.9)	48 (27.9)	
**Education level**			
Illiterate	46 (26.9)	58 (33.7)	0.261
Lower than high school	54 (31.6)	39 (22.7)	
High school	38 (22.2)	42 (24.4)	
University and higher	33 (19.3)	33 (19.2)	
**Residence**			
Rural	90 (52.3)	78 (45.3)	0.196
Urban	82 (47.7)	94 (54.7)	
**Family monthly income** (SAR)			
<5000	69 (40.6)	81 (47.1)	0.591
5000–10000	61 (35.9)	51 (29.7)	
10000–15000	30 (17.6)	31 (18.0)	
>15000	10 (5.9)	9 (5.2)	
	***Median (IQR)***	***Median (IQR)***	***p***
**Age** (years)	46 (17)	47 (0.00)	0.927
**Cigarettes smoked** (per day)	10 (18)	20 (0.00)	0.098*
**Waterpipe heads consumed** (per day)	2 (1)	1.00 (0.00)	0.043
**Khat consumed** (per day)	1 (0.0)	1.00 (0.00)	0.726*
**Clinical characteristics**			
TC (mmol/L)	4.0 (2.58)	3.22 (0.00)	0.000
LDL (mmol/L)	2.39 (2.55)	1.655 (0.00)	0.000
HDL (mmol/L)	0.89 (0.35)	0.93 (0.00)	0.008
TG (mmol/L)	1.76 (2.40)	2.07 (0.00)	0.000
SBP (mmHg)	132 (18)	138.0 (0.00)	0.269
DBP (mmHg)	80 (6)	84.00 (0.00)	0.262
FPG (mmol/L)	6.15 (3.0)	7.20 (0.00)	0.911
Moderate physical activity (per day)	15 (53)	0.00 (0.00)	0.002

TG: triglycerides. TC: total cholesterol. LDL: low-density lipoprotein HDL: high-density lipoprotein. SBP: systolic blood pressure. DBP: diastolic blood pressure. FPG: fasting plasma glucose. IQR: interquartile range.

* Based on Mann-Whitney U test. SAR: 1000 Saudi Riyal about US$270.

**Figure 2:**
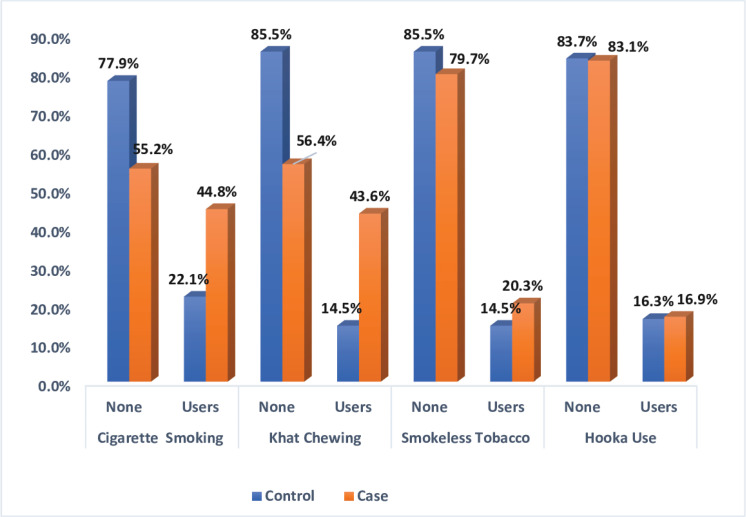
The prevalence of substance abusers among case and control groups, involved in the study of the relationship between the use of Khat and tobacco products and the risk of acute coronary syndrome in the Jazan region of Saudi Arabia, in 2019

Figure 2 shows the prevalence of substance abuse among cases and controls. The prevalence of ever khat chewing was significantly higher for the cases (43.6%) than for the control (14.5%) (p<0.001). Cigarette smokers accounted for 22.1% of the cases, which is a significantly higher percentage than the control group (p<0.001). The prevalence of smokeless tobacco was similar between the two groups (p>0.05).

Table 2 presents the clinical, physical activity, and anthropometric factors associated with ACS (acute coronary syndrome). Among these factors, several showed significant associations with ACS, along with their corresponding adjusted odds ratio (AOR) and 95% confidence intervals (CI). The factors and their respective AOR and confidence intervals were as follows: family history of myocardial infarction (MI) (AOR=2.17; 95% CI: 1.38–3.41, p=0.001), hypertension (AOR=2.82; 95% CI: 1.79–4.42, p<0.0001), moderate physical activity (AOR=0.42; 95% CI: 0.27–0.67, p=0.0001), central obesity (AOR=1.90; 95% CI: 1.15–3.14, p=0.013), high cholesterol (AOR=62.28; 95% CI: 8.47–457.79, p<0.0001), LDL cholesterol (AOR=36.30; 95% CI: 4.90–269.18, p<0.0001), and diabetes (AOR=1.99; 95% CI: 1.14–3.50, p=0.016).

**Table 2:** The clinical, physical activity, and anthropometric factors associated with ACS (acute coronary syndrome) in the case and control groups involved in the study of the relationship between the use of khat and tobacco products and the risk of acute coronary syndrome in the Jazan region of Saudi Arabia, in 2019 (N=344)

*Factors*	*Cases*	*Controls*	*AOR*	*95 % CI*		*p*
	*n (%)*	*n (%)*		*Lower*	*Upper*	
**Family history of MI**						
No	96 (55.8)	126 (73.3)				
Yes	76 (44.2)	46 (26.7)	2.17	1.38	3.41	0.001
**Hypertension**						
No	83 (48.5)	125 (72.7)				
Yes	88 (51.5)	47 (27.3)	2.82	1.79	4.42	<0.0001
**Physical activity**						
Low	113 (65.7)	77 (44.8)				
Moderate	51 (29.7)	82 (47.7)	0.42	0.27	0.67	0.0001
High	8 (4.7)	13 (7.6)	0.42	0.17	1.05	0.066
**Waist–hip ratio** (central obesity)						
Normal	120 (69.8)	140 (81.4)				
Obese	52 (30.2)	32 (18.6)	1.90	1.15	3.14	0.013
**Total cholesterol**						
Normal	114 (69.5)	142 (99.3)				
High	50 (30.5)	1 (0.7)	62.28	8.47	457.79	<0.0001
**Serum triglycerides**						
Normal	63 (38.7)	61 (42.7)				
High	100 (61.3)	82 (57.3)	1.18	0.75	1.87	0.476
**Diabetes mellitus**						
Normal	115 (67.3)	116 (71.2)				
Diabetic	56 (32.7)	47 (28.8)	1.99	1.14	3.50	0.016
**LDL**						
Normal	130 (79.8)	143 (99.3)				
High	33 (20.2)	1 (0.7)	36.30	4.90	269.18	<0.0001

AOR: adjusted odds ratio. MI: myocardial infarction.

Table 3 illustrates the factors associated with tobacco use habits and MI (myocardial infarction). Tobacco use was strongly associated with MI, as evidenced by the significant association between being an ever smoker and MI (AOR=2.86; 95% CI: 1.79–4.57, p<0.0001). Current smoking was also associated with MI (AOR=2.88; 95% CI: 1.65–5.01; p<0.0001). Specifically, cigarette smoking was more likely among those with MI (AOR=23.8; 95% CI: 3.11–181.92; p=0.002), as was waterpipe smoking (AOR=11.3; 95% CI: 1.29–99.23, p=0.028), and the co-use of cigarettes and waterpipe (AOR=23.8; 95% CI: 3.11–181.92, p=0.002).

**Table 3:** The association between the smoking related factors and the risk of acute coronary syndrome in the case and control groups involved in the study of the relationship between the use of khat and tobacco products and the risk of acute coronary syndrome in the Jazan region of Saudi Arabia, in 2019 (N=344)

*Factors*	*Cases*	*Controls*	*AOR*	*95 % CI*		*p*
	*n (%)*	*n (%)*		*Lower*	*Upper*	
**Ever smoke**						
No	95 (55.2)	134 (77.9)				
Yes	77 (44.8)	38 (22.1)	2.86	1.79	4.57	<0.0001
**Current smoke**						
No	95 (66.0)	134 (84.8)				
Yes	49 (34.0)	24 (15.2)	2.88	1.65	5.01	<0.0001
**Type of smoked tobacco**						
Non-smoker	95 (55.2)	134 (77.9)				
Cigarette	45 (26.2)	28 (16.3)	23.80	3.11	181.92	0.002
Waterpipe	15 (8.7)	10 (5.8)	10.58	1.33	83.93	0.026
Cigarette and waterpipe	17 (9.9)	0 (0.0)	11.33	1.29	99.23	0.028
**Duration of cigarette smoking** (years)						
Non-smoker	105 (61.0)	152 (88.4)				
1–10	7 (4.1)	2 (1.2)	5.68	1.83	11.88	0.003
11–20	26 (15.1)	9 (5.2)	4.21	1.88	6.12	<0.0001
>20	34 (19.8)	9 (5.2)	4.42	2.19	3.76	<0.0001
**Cigarettes per day**						
Non-smoker	107 (62.2)	152 (88.4)				
1–10	24 (14.0)	11 (6.4)	3.10	1.46	6.60	0.003
11–20	30 (17.4)	8 (4.7)	5.33	2.35	12.07	<0.001
>20	11 (6.4)	1 (0.6)	15.63	1.99	122.85	0.009
**Smoking cessation** (years)						
<5	9 (32.1)	3 (21.4)				
5–9	7 (25.0)	2 (14.3)	0.44	0.09	2.13	0.310
≥10	12 (42.9)	9 (64.3)	0.38	0.06	2.29	0.292
**Ever use of smokeless tobacco**						
No	137 (79.7)	147 (85.5)				
Yes	35 (20.3)	25 (14.5)	1.50	0.86	2.64	0.157
**Current use of smokeless tobacco**						
No	137 (86.7)	147 (91.3)				
Yes	21 (13.3)	14 (8.7)	1.61	0.79	3.29	0.192
**Number of waterpipe heads consumed**						
Non-user	144 (83.7)	144 (83.7)				
1	10 (5.8)	13 (7.6)	3.50	0.71	17.13	0.122
2	11 (6.4)	13 (7.6)	4.55	0.77	26.84	0.094
≥3	7 (4.1)	2 (1.2)	4.14	0.71	24.16	0.115

AOR: adjusted odds ratio.

Furthermore, the risk of MI varied depending on the number of cigarettes smoked. Patients who smoked 1–10 cigarettes per day had an AOR of 3.10 (95% CI: 1.46–6.60, p=0.0003), 11–20 cigarettes per day had an AOR of 5.33 (95% CI: 2.35–12.07, p<0.001), and >20 cigarettes per day had an AOR of 15.63 (95% CI: 1.99–122.85, p<0.001).

The duration of smoking also plays a role in the association between smoking and MI. For patients who smoked ≤10 years, the AOR was 5.68 (95% CI: 1.83–11.88), 11–20 years, the AOR was 5.33 (95% CI: 2.35–12.07, p<0.001) and >20 years, the AOR was 4.42 (95% CI: 2.35–12.07, p<0.001).

Khat chewing was significantly associated with MI (Table 4). Khat chewing habits and factors that were significantly related to the acute coronary syndrome, their adjusted odds ratio (AOR) and 95% confidence intervals (CI) were: ever khat use (AOR=4.55; 95% CI: 2.70–7.65, p<0.0001); current khat use (AOR=5.41; 95% CI: 2.84–10.32; p=0.0001); chewing khat for the 1–10 years (AOR=5.68; 95% CI: 1.83–17.63, p=0.0026); khat use for 11–20 years (AOR=4.21; 95% CI: 1.88–9.41, p=0.001); and khat use >20 years (AOR=4.42; 95% CI: 2.70–8.94, p=0.0001). MI was significantly associated with the amount of khat consumed per day in hours and weeks (p<0.05 for all). MI was also found to be significantly associated with the co-use of khat with cigarette smoking (AOR=7.36; 95% CI: 3.14–17.27 p<0.0001) (Table 4).

**Table 4:** The association between the khat chewing related factors and the risk of acute coronary syndrome in the case and control groups involved in the study of the relationship between the use of khat and tobacco products and the risk of acute coronary syndrome in the Jazan region of Saudi Arabia, in 2019 (N=344)

*Factors*	*Cases*	*Controls*	*AOR*	*95 % CI*		*p*
	*n (%)*	*n (%)*		*Lower*	*Upper*	
**Ever khat chewer**						
No	97 (56.4)	147 (85.5)				
Yes	75 (43.6)	25 (14.5)	4.55	2.70	7.65	<0.0001
**Current khat chewing**						
No	97 (66.0)	147 (91.3)				
Yes	50 (34.0)	14 (8.7)	5.41	2.84	10.32	<0.0001
**Khat chewing duration** (years)						
Non-user	97 (56.4)	147 (85.5)				
1–10	15 (8.7)	4 (2.3)	5.68	1.83	17.63	0.0026
11–20	25 (14.5)	9 (5.2)	4.21	1.88	9.41	0.0005
>20	35 (20.3)	12 (7.0)	4.42	2.17	8.94	<0.0001
**Khat bundles consumed per day**						
Non-user	97 (56.4)	147 (85.5)				
<1	25 (14.5)	10 (5.8)	3.79	1.70	8.24	0.0008
1	39 (22.7)	11 (6.4)	5.37	2.62	11.00	0.0001
>1	11 (6.4)	4 (2.3)	4.14	1.29	13.64	0.0171
**Khat session** (hours)						
Non-user	97 (56.4)	147 (85.5)				
1–3	26 (15.1)	6 (3.5)	6.6	2.61	16.54	0.0001
4–5	32 (18.6)	13 (7.6)	3.73	1.86	7.46	0.0002
≥6	17 (9.9)	6 (3.5)	4.29	1.63	11.27	0.0031
**Khat cessation** (years)						
<5	11 (42.3)	3 (27.3)				
5–9	8 (30.8)	3 (27.3)	0.73	0.11	4.56	0.272
≥10	7 (26.9)	5 (45.5)	0.38	0.09	2.13	0.472
**Number of khat chewing sessions per week** (days)						
Non-user	97 (56.4)	147 (85.5)				
1–2	31 (18.0)	7 (4.1)	6.17	2.82	15.85	<0.0001
3–4	23 (13.4)	15 (8.7)	2.32	1.15	4.68	0.0181
5–7	21 (12.2)	3 (1.7)	10.60	3.08	36.53	0.0002
**Co-use of tobacco during khat chewing**						
Non-user	97 (56.4)	147 (85.5)				
Khat only	31 (18.0)	11 (6.4)	4.27	2.05	8.90	0.000
Khat and cigarettes	34 (19.8)	7 (4.1)	7.36	3.14	17.27	0.0001
Khat and waterpipe	10 (5.8)	7 (4.1)	3.16	0.80	5.88	0.1289

AOR: adjusted odds ratio.

Table 5 illustrates four logistic regression models. Model 1 describes the classical predictors of MI without any substance-use variables, which are family history of MI, hypertension, moderate and high physical activities, and high cholesterol level. Model 2 shows the contribution of tobacco use as one of the significant confounders of MI. In contrast, Model 3 addresses the contribution of regular khat chewing. Model 4 incorporates both tobacco and khat chewing in the logistic model along with the other classical MI predictors.

**Table 5:** Logistic regression analysis of independent predictors of having an acute coronary syndrome in the study of the relationship between the use of khat and tobacco products and the risk of acute coronary syndrome in the Jazan region of Saudi Arabia, in 2019

*Variables*	*Model 1*				*Model 2*				*Model 3*				*Model 4*			
	*p*	*AOR*	*95 % CI*		*p*	*AOR*	*95 % CI*		*p*	*AOR*	*95 % CI*		*p*	*AOR*	*95 % CI*	
			*Lower*	*Upper*			*Lower*	*Upper*			*Lower*	*Upper*			*Lower*	*Upper*
Family history of MI	0.016	2.01	1.14	3.56	0.029	1.91	1.07	3.43	0.015	2.08	1.15	3.77	0.040	1.98	1.03	3.78
Hypertension	0.003	2.36	1.34	4.16	0.003	2.39	1.34	4.25	0.002	2.56	1.42	4.61	0.008	2.39	1.26	4.53
Moderate physical activity	0.038	0.54	0.31	0.94	0.035	3.49	1.09	11.15	0.055	0.56	0.31	1.00	0.033	0.50	0.26	0.94
High physical activity	0.317	0.30	0.09	0.93	0.244	2.03	0.62	6.64	0.373	0.33	0.10	1.02	0.029	0.26	0.08	0.87
High cholesterol level	0.000	2.17	1.71	2.75	0.000	2.16	1.70	2.75	0.000	2.17	1.69	2.80	0.000	2.27	1.71	3.01
Diabetes	0.028	1.94	1.07	3.52	0.056	1.81	0.98	3.34	0.020	2.10	1.12	3.92	0.089	1.81	0.91	3.60
Regular tobacco use					0.002	2.54	1.41	4.58					0.048	4.58	1.01	4.73
Regular khat use									0.000	4.42	2.29	8.50	0.002	3.40	1.55	7.46

Mosel 1: without any substance use. Model 2: with regular tobacco use. Model 3: with regular khat chewing. Model 4: with tobacco use and khat chewing. AOR: adjusted odds ratio; adjusted for selected variables in the table. Low physical activity is the reference for moderate and high physical activity. MI: myocardial infarction.

Model 1 highlighted a significant association between MI and all predictors in the model, except for a high level of physical activity. In Model 2, MI was associated with regular tobacco use (AOR=2.54; 95% CI: 1.14–4.58, p<0.001), adjusting for other classical risk factors of MI. Model 3 demonstrated that MI was associated with regular khat chewing (AOR=4.42; 95% CI: 2.29–8.50, p<0.0001), controlling for other classical MI risk factors presented in the model. In Model 4, MI was associated with both tobacco use and khat chewing after controlling for other classical risk factors outlined in the model. The adjusted odds ratio for tobacco use was 4.58 (95% CI: 1.01–4.73, p<0.05), and for khat chewing, it was 3.4 (95% CI: 1.55–7.46, p<0.05).

## DISCUSSION

This study examined the risk factors related to cardiovascular diseases in the Jazan region of Saudi Arabia. It is the first study in the country to explore the link between khat consumption, tobacco use, and ACS risk. Prevalent risk factors included smoking, hypertension, diabetes, hypercholesteremia, central obesity, and family history of MI, all significantly associated with ACS^3,4^. Hypercholesteremia and smoking were particularly prevalent. Moderate physical activity, such as daily 30-minute walks for five days or more, showed a protective effect against ACS, consistent with prior research^22-24^. The study did not find a significant association between advanced age, male sex, and ACS, potentially influenced by the age composition of the study population, with two-thirds aged ≥50 years and women likely in the menopause stage^25^.

Chewing khat is considered socially acceptable behavior among the Jazan population. The overall prevalence of khat chewing was 29.1% among the study population, which is, according to the available literature data, similar to what was reported in the Jazan region^7,14^. In our case-control study, khat chewing was three times higher among cases than controls, with almost half of the ACS cases being khat consumers.

There has been limited research on the association between khat chewing and ACS. A study in Yemen found that almost 80% of MI patients were khat chewers, and over 70% of MI events occurred during or immediately after khat chewing^26^. Our most important finding was that the odds of khat chewing were significantly associated with ACS. People who had ever chewed khat were 4.5 times more associated with ACS than people who had never chewed khat, and current khat chewers were 5.4 times more associated with ACS. Even after controlling for other classical MI risk factors in logistic regression models, regular khat chewing was independently associated with ACS.

The association between khat consumption and myocardial infarction (MI) observed in this study aligns with a similar survey from a previous study. A case-control study conducted in Yemen found that khat consumers were five times more likely to experience an acute MI^26-29^. Furthermore, that study demonstrated a dose-response relationship, with higher quantities of khat significantly associated with MI. However, our study did not identify a dose-response relationship between khat and MI, primarily due to the difficulties in estimating the quantity of khat consumed. The number of leaves in a bundle of khat can vary widely, and the varying quality of khat leaves makes only fresh leaves suitable for chewing^27^. Additionally, the social nature of khat sessions often involves sharing khat leaves among participants, making it challenging to accurately quantify khat consumption^7^.

The potential mechanisms linking khat to ACS can be explained by its amphetamine-like mode of action^8^. Amphetamine abuse has been consistently associated with the occurrence of acute adverse cardiac events in various reports and clinical studies. Cathinone, the primary active substance in khat, is known to stimulate the release of catecholamines. This, in turn, raises heart rate and blood pressure, increases cardiac oxygen demand, constricts coronary circulation, and triggers catecholamine-induced platelet activation and aggregation. These effects ultimately contribute to the risk of ACS. The significant increase in blood pressure caused by cathinone can also induce stress and disrupt coronary atherosclerotic plaque, further increasing the likelihood of ACS development^8,29^.

Another significant finding in this study was that one in five khat consumers reported using cigarettes while chewing khat. This observation aligns with findings from two similar studies that also reported a higher prevalence of cigarette smoking among khat chewers, with one study even documenting a 60% prevalence. The concurrent use of tobacco products and khat may increase the risk of ACS, potentially acting synergistically^7,30^.

For tobacco products, our study found a statistically significant association of ACS with cigarette smoking and waterpipe use but no significant association with smokeless tobacco chewing.

The study revealed a dose-response relationship between cigarette consumption and ACS. Those who smoked 20 cigarettes per day had five-fold higher odds associated with ACS compared to those who smoked ≤10 cigarettes per day. However, establishing a dose-response relationship between waterpipe smoking and ACS proved challenging due to the subjective nature of measuring waterpipe use. Waterpipes involve various substances and chemical structures, and the amount of nicotine produced can vary significantly based on factors like heating temperature, tobacco mixture, puffing behavior, and waterpipe characteristics^10,31^. Consequently, accurately quantifying waterpipe use becomes subjective, making it difficult to establish a clear dose-response relationship13,15,31.

Cigarette smoking is a well-documented risk factor for various cardiovascular diseases, including atherosclerotic coronary artery disease, peripheral vascular disease, cerebrovascular disease, and abdominal aortic aneurysm, affecting blood vessels of different sizes^15,32^. Smokers face a higher risk of recurrent MI and sudden cardiac death, while quitting smoking significantly reduces the risk of severe coronary events^33^.

Numerous studies have demonstrated the adverse cardiovascular effects of tobacco smoking, particularly cigarettes. Components such as free radicals, nicotine, toxic gases, and carbon monoxide in tobacco smoke induce oxidative stress and vascular inflammation, leading to endothelial dysfunction. This dysfunction initiates the development of atherosclerosis^34^. Other mechanisms include vascular inflammation, platelet coagulation, alteration of antithrombotic and prothrombotic factors, arterial stiffness, and the hypertensive effects of nicotine-induced sympathetic activation. These processes accelerate the progression of atherothrombosis, culminating in acute adverse coronary events. While this pathophysiology is more prominent in cigarette smoking, it also applies to some extent to waterpipe smoke but is less likely to occur with other tobacco products^31,34^.

Waterpipe smoking has gained popularity among young adults, with over half of waterpipe smokers being under 25 years old^10^. While some studies have reported an association between long-term waterpipe smoke usage and an increased risk of coronary artery disease, others have not observed an elevated risk of cardiovascular disease (CVD). However, our casecontrol study demonstrated a statistically significant link between waterpipe smoke and an increased risk of ACS. A similar study conducted in Saudi Arabia’s Al-Madinah region found a higher risk of MI in individuals who had never smoked waterpipe before, with an even greater risk among exclusive waterpipe smokers^31^. A systematic review investigating the effects of waterpipe use on cardiovascular health showed that waterpipe smokers had higher in-hospital mortality rates and a higher occurrence of ST-elevation myocardial infarction (STEMI) compared to non-ST-elevation myocardial infarction (NSTEMI) among cigarette smokers. Long-term waterpipe users also exhibited a worse atherosclerotic risk profile and an increased risk of recurrent MI^11^.

The relationship between smokeless tobacco use and the risk of ACS remains a topic of debate^31^. This study’s findings on smokeless tobacco align with previous studies that did not establish a clear association between smokeless tobacco use and adverse cardiovascular outcomes. Recent systematic reviews and meta-analyses have shown varying results on the association between smokeless tobacco use and circulatory disease risk, with variations observed across different regions^11,35^. For instance, Asian countries have demonstrated an association between smokeless tobacco use and the risk of non-fatal acute coronary events, while European countries have not established a clear and significant association^36^. Limited data exist on smokeless tobacco use and cardiovascular risk in Middle Eastern countries, particularly Saudi Arabia, necessitating further investigation to better understand this relationship.

### Strengths and limitations

While this observational study provides valuable insights into ACS risk factors among our population, it has several limitations. First, it cannot establish a causal relationship. Second, recall bias may be present due to the case-control design. Third, the study did not fully control for all potential confounding factors, such as dietary patterns and psychological factors. Fourth, subjective measurement of khat and waterpipe consumption may have affected the dose-response relationship. Fifth, the study did not define the specific types of waterpipe and smokeless tobacco used, despite the significant heterogeneity in the quality and composition of these products. On the positive side, the study’s strengths include a prospective design, inclusion of newly incident cases to minimize recall bias, standardized questionnaires, and data collection through interviews by trained nurses, ensuring accurate data and high response rates. These findings will contribute to the implementation of early preventive measures and risk modification interventions aimed at reducing cardiovascular events in our region.

## CONCLUSIONS

The common population-attributable risk factors for acute coronary syndrome (ACS) in the southwestern region of Saudi Arabia include cigarette smoking, waterpipe smoking, hypertension, diabetes mellitus, hypercholesterolemia, central obesity, khat chewing, and a family history of myocardial infarction (MI). ACS was associated with cigarettes and waterpipe smoke, as well as khat use in the Jazan region. Future intervention programs are needed to address these risk factors for the primary and secondary prevention of adverse coronary events in the Jazan population.

## Data Availability

The data supporting this research are available from the authors on reasonable request.
